# In vivo investigation of female reproductive functions and parameters in nonpregnant mice models and mass spectrometric analysis of the methanol leaf extract of *Emilia Coccinea* (Sims) G Dons

**DOI:** 10.14814/phy2.13047

**Published:** 2016-12-16

**Authors:** Uloma B. Elvis‐Offiah, Enitome E. Bafor, Gerald I. Eze, Osamwonyi Igbinumwen, Christina Viegelmann, RuAngelie Edrada‐Ebel

**Affiliations:** ^1^ Department of Science Laboratory TechnologyFaculty of Life SciencesUniversity of BeninBenin CityNigeria; ^2^ Department of Pharmacology and ToxicologyFaculty of PharmacyUniversity of BeninBenin CityNigeria; ^3^ Department of AnatomySchool of Basic Medical SciencesUniversity of BeninBenin CityNigeria; ^4^ Haematology DepartmentUniversity of Benin Teaching HospitalBenin CityNigeria; ^5^ Strathclyde Institute of Pharmacy and Biomedical SciencesGlasgowUK

**Keywords:** Contraception, *Emilia coccinea*, female reproductive cycle

## Abstract

In Southern Nigeria, the leaves of *Emilia coccinea* (Sims) G Dons are used traditionally for birth control. This study was therefore aimed at evaluating the activities of the methanolic leaf extract of *Emilia coccinea* (EM) on parameters that affect reproduction as well as the acute toxic effects of the plant using nonpregnant female mice models. Leaves of EM were extracted by maceration with 99.8% methanol. Oral acute toxicity profiles were examined. The effects of EM on female reproductive cycle were determined after oral treatment with EM at 1000 and 100 mg/kg/day daily for 6 days using stilbesterol (1 mg/kg/day) and normal saline as controls. The activities of EM (1000 mg/kg/day and 100 mg/kg/day p.o) on reproductive hormones and organs were also studied using estradiol valerat (100 mg/kg/day p.o), progesterone (10 mg/kg/day s.c.), and normal saline as controls. The extract did not induce any observable toxic effect after 24 h. At 1000 mg/kg, the extract significantly shortened the estrus cycle (*P* < 0.05) while prolonging the estrus phase which were comparable to that observed with stilbesterol. The extract also increased uterine weight and altered the histology of uterine and ovarian tissues. The female reproductive hormones were additionally altered at 1000 mg/kg and the effects were comparable to that of estradiol valerat such as to indicate possible antifertility effects. LC‐HRFTMS analysis showed 9 putatively identified compounds with pyrrolizidine alkaloid occurring at the highest intensity among the identified compounds. In conclusion, the leaf extracts of EM has been shown in this study to exhibit antiovulatory and estrogenic activities which would support the traditional use of the plant in Nigeria.

## Introduction

For centuries, the use of plants to maintain and manage human health and wellbeing has been a common practice among the African communities. To date, about 90% of the African population still depends solely on this practice (Cunningham 1993). Some of these preparations have, however, been associated with toxic effects which can lead to mortality or morbidity therefore necessitating careful monitoring.

*Emilia coccinea* (Sims) G Dons (EM) known as “yellow tassel flower” in the English language is an edible plant commonly used in traditional medicine within tropical parts of Africa such as Nigeria for birth control. *E. coccinea* is of the family Compositae (Asteraceae) (Olorode 1984). It is considered and described as a ubiquitous weed of the waste place and fallow land, widely distributed in tropical Asia and in tropical rain forests of West Africa (Edeoga et al. 2005; Chillendon 1956).

Phytochemical compounds such as alkaloids, terpenoids, flavonoids, tannins, saponins, steroids, and cardiac glycosides have been previously reported in EM (Edeoga et al. 2005; Okiei et al. 2009; Idu et al. 2010; Sofowora 1982; Teke et al. 2007; Mensah et al. 2013). These constituents are known to have medicinal and varied physiological effects in humans (Sofowora 1993; Kubmarawa et al. 2007; Addae‐Mensah 1999; Okoegwale and Omefezi 2001; Okoegwale and Olumese 2001; Ogunlesi et al. 2008). Previous studies also reported EM to possess antioxidant, antimicrobial, antidiabetic, anti‐inflammatory, antidiarrhea, anxiolytic, antidepressant as well as antihepatotoxic activities (Erhabor et al. 2013; Agoha 1981; Burkill 1984; Odugbemi 2006; Telefo et al. 2011; Foyet et al. 2014).

However, no scientific data or reports are available on the activity of the plant on the female reproductive system. This study is therefore aimed at investigating potential antifertility properties of the plant by examination of the plant effect on the female reproductive cycle, organs, and hormones using mouse models. This is necessary to provide a proof of concept for the use of the plant as a contraceptive by traditional healers in Nigeria. The acute toxicity effects of the plant were also investigated in this study in order to determine the safety profile of the plant.

The choice of animal model has great impact on the outcome of results. The mice model is an established model to study effects of agents on humans as they are easy and flexible to handle and manipulate (Groothuis et al. 2007). Many authors have shown that similarities exist between mouse and human reproductive organs and cycle (Nelson et al. 1981; Kurita et al. 2005) thus, allowing careful extrapolation of findings to human. The reproductive cycles of both mice and humans are under the control of the endocrine system and are responsible for reproduction. The reproductive cycle is an endocrine clock that transmits time through regular periodic fluctuations in the concentration of mean values of reproductive hormones of the hypothalamus, anterior pituitary, and ovaries and transduce the information in a timely dependent manner to the uterus and ovaries. Mice models have been used to demonstrate the roles of two estrogen receptor (ER) isoforms in endometrial regulation (Hewitt et al. 2005, 2003).

## Materials and Methods

### Plant samples collection and extract preparation

Fresh samples of *Emilia coccinea (Sims) G Dons* leaves were collected in July within the locality of the Obingwa Local Government Area of Abia State, Nigeria between 6.00 a.m and 9.00 a.m and authenticated by Dr. H. A. Akinnibosun of the Department of Plant Biology and Biotechnology, University of Benin, Nigeria. The herbarium sample with voucher number, UBH_a_302 was prepared and deposited for future references. The leaves were then cleaned and dried for 10 days at room temperature 24–26°C.

The dried samples were blended into powder with the aid of an electric blender. The powdered sample was then macerated in 100% methanol and stirred repeatedly for 24 h. After the 24 h maceration process, the macerate was filtered through a Whatman No. 1 filter paper. The residue was discarded and the filtrate was evaporated to dryness using a water‐bath set at 60°C. The concentrate was further dried to a constant weight in a Hotbox oven (Gallenramp^®^, England) set at 40°C. The dried extract was kept in a refrigerator until usage. The given powder yielded 11.35% of a dark green extract (EM).

### Animals used

Adult female Swiss Albino mice (25–35 g) aged 3–4 months, were used in all experiments. The animals were bought from a local Animal Center in Benin City, Nigeria, and kept in the Animal House of the Department of Pharmacology and Toxicology, University of Benin, Nigeria for the duration of the experiments. The mice were handled according to standard guidelines for the use and care of Laboratory experimental animals as stated by the Ethical committee, Faculty of Pharmacy, University of Benin, Nigeria as well as the standard guidelines for use of laboratory animals (National Institute of Health, Bethesda, MD: Public Health Service Policy on Humane Care and Use of Laboratory animals, 2002) and acclimatized for 2 weeks before the experiment proper.

### Acute toxicity study

The oral acute toxicity study was carried out according to methods of Miller and Tainter (Miller and Tainter 1944). The animals were randomly divided into five (5) groups of five (5) mice each for the treatments. A single oral dose of 2000, 1000, 500, or 100 mg/kg of the extract was administered to groups A–D, respectively, by means of an orogastric tube connected to a syringe. Group E orally received distilled water (1 mL/kg) and served as the control. The animals in all the groups were observed during the first 4 h after the single oral administration of the extracts for behavioral, neurological, and autonomic profiles and for any lethality or death up to 24 h after administration. These include locomotion, reaction to noise, tail activities and the appearance of feces. After the first 4 h of observation, all the animals were allowed free access to food and water. The surviving animals after 24 h were sacrificed and their blood samples were collected with 27G needles on 1 ml syringe into EDTA and lithium heparinzed bottles for hematological and biochemical tests, respectively.

The biochemical analysis was performed on serum obtained after centrifugation of whole blood (without anticoagulant) at 1680 *g* for 5 min. Standardized diagnostic kits (Randox^®^ by Randox Laboratories LTD, Crumlin, UK) were used for spectrophotometric (UNICO^®^ S1200 spectrophotometer, UNICO Suite E, Dayton, NJ) determination of the following biochemical parameters: alanine aminotransferase (ALT), aspartate aminotransferase (AST), creatinine, urea, biocarbonate, total proteins, albumin, total and conjugated bilirubin, sodium, chloride, and potassium. ALT and AST were analyzed by adopted method of Reitman and Frankel (1957). The biuret method of protein estimation of Kaplan (1972) was used in the assay for the total amount of protein in each sample. The assay for the total amount of albumin present in each sample was carried out using a method adopted from Young et al. (1975) using bromocresyl purple dye. And the assay for the total amount of bilirubin and the amount of conjugated bilirubin present in each sample was estimated by the adopted method of Jendrassik and Grof (1938).

The hematological parameters were analyzed at the University of Benin Teaching Hospital (UBTH), Nigeria using Sysmex (Kx‐21N) automated hematological analyzer for in vitro diagnostic use in clinical laboratories. The samples were manually diluted with a diluting factor of 1–200 before loading into the automated hematological analyzer.

### The effect of EM on ovulation/female reproductive (Estrus) cycle

The stages of the estrus cycle for each mouse was determined daily between 9 a.m and 12 Noon. The daily determinations were performed for 2 weeks (14 days) by both the visual method (Macroscopic Observation) and the swab method (microscopic observation) of obtaining vaginal smears (Snell 1941). Both procedures were performed independently. Animals found to be pseudopregnant were excluded from the experiments. Animals with similar cycles were placed together in a group.

In the Visual Examination (Macroscopic) method of observation, a modified method of Elvis‐Offiah and Bafor (2014) was employed. This method utilized the changes in the appearance of the vagina that occur during the estrus cycle; the degree of vaginal swelling (particularly with respect to the dorsal lip), the color, and moistness were examined. The size of the vaginal opening and the presence or absence of cellular debris in the vagina was also examined. The mammary glands were observed to detect changes in appearance. The abdominal distension was also measured in order to ascertain the possible size of the uterus.

The Swabbing Method of Vaginal Smear Examination (Microscopic Observation) utilized a slight modification of the technique of Stockard and Papanicolaou (1917) and it basically involved identification of cell types and their relative quantities present in the preparation obtained from the vagina by swabbing the vaginal walls. Attributions of these characteristics to specific cycles were ascribed and scored as described by Elvis‐Offiah and Bafor (2014).

Vaginal smears were collected from the animals daily by flushing the vagina with distilled water using a micropipette. The mouse was placed in a position which reduced animal's movement as much as possible and the swabbing pipette was introduced gently at a depth of approximately 0.1 cm into the mouse vagina. The swab pipette was released to flood the vaginal epithelium and a small quantity of vaginal fluid was drawn. The fluid containing the cells was gently transferred onto a clean, prelabeled glass slide, below the relevant animal number. Care was taken to minimize pseudopregnancy. The smears were fixed with 95% ethyl alcohol and stained with Gentian Violet (GV) for 2 min. The GV was then gently rinsed off with water and allowed to dry. The smears were observed under LABO Optical Binocular Microscope, model AXL, (Haryana, India) attached to digital camera (1.3 M pixel) connected to a computer using ×10 magnification.

### Extract administration

After complete estrus cycles of the animals were ascertained, the animals with similar estrus cycles were placed in the same group of five animals per group, whereas animals found pseudopregnant were eliminated. Group I animals were in their proestrus phase and were given stilbesterol (1 mg/kg p.o.) thus serving as the positive control, Groups II (diestrus phase) and IV (metestrus phase) were given 1000 mg/kg (p.o.) and 100 mg/kg (p.o.) of the extract, respectively, whereas Group III (estrus phase) were given normal saline (p.o) and served as the negative control. The administration was carried out once daily for 6 days (6 days had been previously determined to cover one complete regular estrus cycle). Macroscopic and microscopic observations were carried out for the 6 days of treatment and subsequently for 10 days after treatment.

### Determination of the activity of EM on hematological parameters and on reproductive cycle, hormones, and organs

Five experimental groups were used for this study. Group I was treated with estradiol valerat 100 mg/kg (p.o.), Groups II and III were treated with EM (1000 and 100 mg/kg p.o.) of the extract, Group IV was treated with progesterone 10 mg/kg (s.c.), whereas Group V was given normal saline (p.o) and served as the negative control. All the treatments were carried out for 6 days. On the 7th day, the animals were quickly sacrificed under anesthesia, and their blood samples collected with 27G needles on 1 mL syringe into lithium heparinzed bottles for hormonal assay. The uteri were also carefully dissected out, surrounding tissues removed, then rapidly blotted on filter papers and quickly weighed on a sensitive balance. The uterine tissues were then fixed in 10% formalin and submitted for histological observations.

Histological examination was carried out in order to evaluate the effect of EM on the gross morphology of the whole uterus. The tissue sections were processed with an automated tissue processor (Citadel, 2000 Fischer Scientific, Loughborough, UK) and embedded in paraffin. Sections of 6 *μ*m thicknesses were cut with a rotary microtome (LEICA RM2235; Leica Biosystems Inc., Buffalo Grove, IL) and stained with hematoxylin and eosin (HE) for morphology assessment. Histological sections were examined using the light microscope (×100) and digital images were taken.

The hormonal assay was done by an automated qualitative test utilizing the Minividas Analyzer, (VIDAS Kit, France) on serum or plasma (lithium heparin), using the Enzyme Linked Fluorescent Assay (ELFA) technique (Yolken and Stopa 1979). Briefly, the assay combines an enzyme immunoassay sandwich method with ELFA. The Solid Phase Receptacle (SPR^®^) served as the solid phase as well as the pipetting device for the assay. Reagents for the assay were ready‐to‐use and predispensed in a sealed reagent strip. The results were automatically calculated using calibration curves (4‐parameter logistics model) and then printed out.

The assay procedure for all the hormones was the same but reaction and testing time varied. The assay for luteinizing hormone (LH), and follicle‐stimulating hormone (FSH) was completed within approximately 40 min; the progesterone assay was completed within 45 min while that for estrogen was completed within 1 h.

### LC‐HRFTMS identification of constituents in extract

LC‐HRFTMS analysis was performed on a Dionex UltiMate‐3000 (DIONEX, Sunnyvale, CA) coupled to a Thermo Scientific Exactive Orbitrap system (Thermo Fisher Scientific (Bremen) GmbH, Bremen, Germany). The column used was an ACE 5 C18 75 × 3.0 mm column from Hichrom Ltd., Reading, UK. Compounds were eluted with a flow rate of 300 *μ*L/min using water (A) and acetonitrile (B), both of which contained 0.1% formic acid, by a gradient starting with 10% B and increasing to 100% B in 30 min. The mobile phase was maintained at 100% B for 5 min after which the column was equilibrated with 10% B. The files were sliced into positive and negative datasets using ProteoWizard (Kessner et al. 2008) prior to data mining using MZmine 2.10 (Pluskal et al. 2010). Peak detection was accomplished using the centroid mass detector and a noise level of 1000. The chromatogram builder generated peak lists from the mass lists obtained from the previous step. The minimum time span was 0.2 min, minimum height was 10,000, and the *m/z* tolerance was set to 0.0001 *m/z* or 5 ppm. Chromatogram deconvolution was accomplished using the local minimum search algorithm with the following parameters: threshold (90%), search minimum in RT range (0.4 min), minimum relative height (5%), minimum absolute height (10,000), minimum ratio of peak top/edge (2), and peak duration range (0.2–5.0 min). The peak lists were deisotoped using the isotopic peaks grouper with an *m/z* tolerance of 0.001 *m/z* or 5 ppm, retention time tolerance of 0.1 minutes (absolute), and maximum charge of 2. The representative isotope was the most intense. The peak lists were then merged using the Alignment function. The weight for *m/*z and for RT was 20, and the RT tolerance was 5%. The aligned peak lists were gap‐filled using the Peak Finder, with an intensity tolerance of 1% and RT tolerance of 0.5 min (absolute). The adducts were identified, together with other complexes that may have formed. The chemical formulas of each peak were predicted using the formula prediction tool developed by MZmine. An algorithm was employed to use the molecular formula data set from Dictionary of Natural Products^®^ (June 2015). The database is customized to be curated and embedded into the MACRO. The monoisotopic exact masses for each metabolite were then calculated to be used for the customized library. The processed data from MZmine was incorporated into the customized library through the built‐in Excel macro for peak identification and dereplication. “Hits” and unidentified peaks were double checked against the MS raw data in Xcalibur 2.2. Hits from the database were accessed using ChemBioFinder version 13 (PerkinElmer Informatics, Cambridge, UK).

### Statistical analysis

All data shown were expressed as mean ± standard error (SEM) and “*n*” represents the number of samples per animal. Significance was evaluated using an appropriate *t*‐test, and where necessary, one‐way analysis of variance followed by Tukey's multiple range tests. *P *≤ 0.05 was considered statistically significant in all cases.

## Results

### Acute toxicity studies

The results of the acute toxicity study of EM in mice after 24 h administration showed no visible signs of toxicity in the animals (Tables 1, 2). Although there were slight increases in some hematological and biochemical parameters, they were considered statistically insignificant when compared with the control. No mortality, neurological, autonomic and behavioral changes were recorded within the dose range up to 2000 mg/kg body weight.

**Table 1:** Effect of EM on hematological parameters after 24 h

Parameters	Group 1 (2000 mg/kg)	Group 2 (1000 mg/kg)	Group 3 (500 mg/kg)	Group 4 (100 mg/kg)	Group 5 (Control)
WBC × 10^3^/*μ*L	9.52 ± 1.45	10.67 ± 1.51	11.60 ± 1.88	8.74 ± 2.35	5.60 ± 1.41
LY × 10^3^/*μ*L	6.28 ± 0.92	7.37 ± 1.44	8.16 ± 1.53	5.92 ± 1.77	3.62 ± 0.93
MO × 10^3^/*μ*L	1.26 ± 0.22	1.2 ± 0.15	1.44 ± 0.17	0.96 ± 0.26	0.74 ± 0.18
GR × 10^3^/*μ*L	1.98 ± 0.5	2.07 ± 0.23	2.00 ± 0.22	1.80 ± 0.39	1.26 ± 0.45
LY %	65.8 ± 3.70	67.83 ± 3.94	69.04 ± 2.30	66.98 ± 2.16	65.72 ± 3.65
MO %	13.14 ± 0.65	11.4 ± 0.44	12.94 ± 0.83	10.82 ± 0.18	13.24 ± 1.19
GR %	21.06 ± 3.06	20.77 ± 3.84	18.02 ± 1.49	22.20 ± 2.06	21.04 ± 4.27
RBC × 10^6^/*μ*L	9.65 ± 0.69	11.04 ± 0.51	10.74 ± 0.38	9.00 ± 1.38	7.55 ± 1.25
Hgb g/dL	12.5 ± 0.74	14.6 ± 1.08	13.72 ± 0.70	12.96 ± 0.81	10.12 ± 1.50
HCT %	38.24 ± 2.2	47.43 ± 5.95	41.86 ± 1.98	41.80 ± 3.74	29.26 ± 5.0
MCV fl	38.38 ± 0.88	42.63 ± 3.39	38.90 ± 0.87	38.00 ± 1.77	38.70 ± 1.36
MCH pg	12.92 ± 0.79	13.13 ± 0.38	12.68 ± 0.30	11.84 ± 0.63	14.08 ± 2.35
MCHC g/dL	32.78 ± 1.57	31.07 ± 1.51	32.72 ± 0.26	31.26 ± 1.09	37.42 ± 8.06
PLT × 10^3^/*μ*L	383.2 ± 177.7	501.33 ± 156.1	624 ± 92.7	454 ± 145	211 ± 79.5

Values are expressed as mean ± SEM (*n* = 5); control group received 0.5 mL/kg normal saline. Although there are slight increases in blood hematological parameters, however, they are considered statistically insignificant when compared with the control.

WBC, white blood cells; LY, lymphocytes; MO, monocytes; GR, granulocytes; RBC, red blood cells; Hgb, hemoglobin; HCT, hematocrit; MCV, mean cell volume; MCH, mean cell hemoglobin; MCHC, mean cell hemoglobin concentration; PLT, platelets; EM, *Emilia coccinea*.

**Table 2:** Effects of EM on biochemical blood plasma parameters of mice after 24 h

Parameters	Group 1 (2000 mg/kg)	Group 2 (1000 mg/kg)	Group 3 (500 mg/kg)	Group 4 (100 mg/kg)	Group 5 (Control)
Total bilirubin (mg/dL)	0.34 ± 0.04	0.38 ± 0.05	0.34 ± 0.06	0.28 ± 0.04	0.36 ± 0.10
Conjugated bilirubin (mg/dL)	0.125 ± 0.025	0.140 ± 0.024	0.12 ± 0.02	0.10 ± 0.00	0.20 ± 0.04
Total protein (g/dL)	4.98 ± 0.30	4.82 ± 0.24	4.42 ± 0.46	3.96 ± 0.33	3.70 ± 0.34
Albumin (g/dL)	3.96 ± 0.33	2.42 ± 0.19	3.96 ± 0.78	2.58 ± 0.38	4.68 ± 0.62
AST (IU/dL)	21.4 ± 1.99	17.6 ± 1.86	29.0 ± 1.52	22.0 ± 2.70	20.4 ± 1.03
ALT (IU/dL)	16.2 ± 1.99	12.8 ± 1.46	19.8 ± 2.42	15.6 ± 1.72	15.6 ± 0.98
Urea (mg/dL)	33.4 ± 1.4	27.8 ± 2.24	37.8 ± 2.44	34.2 ± 3.09	35.8 ± 1.83
Sodium (mMol/L)	72.6 ± 4.76	88.6 ± 7.64	59.6 ± 10.27	58.8 ± 8.74	47.0 ± 2.24
Potassium (mMol/L)	3.56 ± 0.17	3.68 ± 0.18	3.32 ± 0.10	3.80 ± 0.19	3.18 ± 0.07
Bicarbonate (mMol/L)	19.8 ± 0.58	19.0 ± 0.63	19.8 ± 1.24	18.4 ± 0.51	18.6 ± 0.6
Chloride (mMol/L)	21 ± 1.0	21.8 ± 1.07	22.6 ± 1.54	20.6 ± 0.87	20.6 ± 0.81
Creatinine (mg/dL)	0.80 ± 0.045	0.66 ± 0.09	0.82 ± 0.09	0.72 ± 0.107	0.88 ± 0.04
Urea (mg/dL)	33.4 ± 1.4	27.8 ± 2.24	37.8 ± 2.44	34.2 ± 3.09	35.8 ± 1.83

Values are expressed as mean ± SEM (*n* = 5 mice); control group received 0.5 mL/kg normal saline. Although there are slight increases in some blood biochemical parameters, however, they are considered statistically insignificant when compared with the control.

AST, aspartate aminotransferase; ALT, alanine transaminase; EM, *Emilia coccinea*.

### Studies on the reproductive cycle

**Figure 1:**
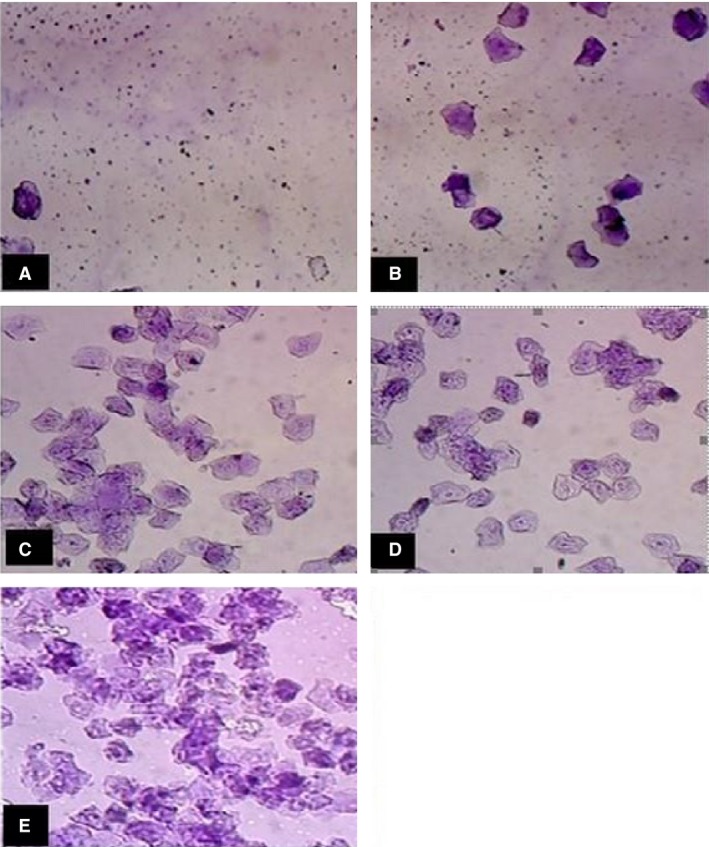
Representative of mouse vaginal smears on 5‐days estrus cycle. LABO AXL binocular microscope ×100. EM (1000 mg/kg)_,_ day 1 (A), day 2 (B), day 3 (C), day 4 (D), day 5 (E). EM, *Emilia coccinea*.

The results of the effect of EM on the female reproductive cycle were examined in several ways. The number of metestrus periods experienced was taken as a measure of the number of cycles completed during the observation. From the macroscopic (visual) and the microscopic (vaginal smear) methods, it was observed that before drug administration the animals had a range of 5–6 day estrus cycles. Four types of cells were distinguished in the vaginal smears, namely: leukocytes, non‐nucleated epithelial cells, nucleated epithelial cells, and cornified cells. The presence and absence of these cell types, and the relative proportion of each cell type, was used to determine the stage of the estrus cycle. The treatment of mice with 1000 mg/kg of the extract for six (06) days, significantly prolonged the estrous cycle (*P* < 0.05) with an accompanying prolonged duration of the estrus phase with 90% cornification of vaginal wall cells (Fig. 1) and shortened duration of the diestrus phase and metestrus phases; whereas the 100 mg/kg dose showed a prolonged metestrus phase (Fig. 1) compared with the control. The effects seen with the higher dose (1000 mg/kg) were similar to the effects observed with the standard drug (stilbesterol, 1 mg/kg), (Table 3). Withdrawal of the treatment did restore the four phases of the estrus cycle and the duration of the cycle.

**Table 3:** Effect of em on ovulation/female reproductive (estrus) cycle

Treatment	Dose (mg/kg)	Length of cycles (days)	Length of different stages of estrus cycle (days)			
			Proestrus	Estrus	Metestrus	Diestrus
Control	—	4.50 ± 0.20	1.00 ± 0.00	1.00 ± 0.20	1.50 ± 0.20	1.00 ± 0.20
EM	1000	6.50 ± 0.2*	1.10 ± 0.20	5.02 ± 0.49**	0.20 ± 0.2	0.18 ± 0.25
EM	100	4.50 ± 1.18	0.80 ± 0.25	1.20 ± 0.29	1.50 ± 0.28	1.00 ± 0.26
Estrogen	1	5.80 ± 0.58*	0.50 ± 0.24	4.00 ± 0.49**	1.0 ± 0.22	0.30 ± 0.10

Values are expressed as mean ± SEM (*n* = 5 mice). Control group received 0.5 ml/kg normal saline.

*P*‐value *(*P* < 0.05) was considered to be statistically significant when compared with the control using one‐way analysis of variance (ANOVA), **P < 0.01. EM, *Emilia coccinea*.

### Effect of EM on reproductive hormones and organs

EM was further studied for activities on some female reproductive hormones and organs. EM induced a significant increase (*P* < 0.01) in uterine weight at 1000 mg/kg (Table 4) without any significant change observed at the lower dose.

**Table 4:** Effect of EM on mouse uterine weight (g)

Groups	Dose (mg/kg body weight)	Weight of Uterus (g)	Weight of Uterus (mg/100 g body weight)
Estradiol valerat	100	0.226 ± 0.028*	10.4 ± 0.001*
EM	1000	0.399 ± 0.10**	14.3 ± 0.036**
EM	100	0.203 ± 0.03	9.0 ± 0.001
Progesterone	10	0.195 ± 0.02	8.4 ± 0.001
Control	—	0.131 ± 0.02	5.5 ± 0.001

Values are expressed as mean ± SEM (*n* = 5 mice). Control group received 0.5 ml/kg normal saline.

*P*‐value *(*P* < 0.05) was considered to be statistically significant when compared with the control using one‐way analysis of variance (ANOVA). **P < 0.01. EM, *Emilia coccinea*.

### Effect of EM on the female reproductive hormones

The assay of the female reproductive hormones indicated that the lower EM dose of 100 mg/kg lacked significant effect on the hormones (Table 5) except for progesterone where a significant reduction (*P* < 0.05) was observed. At the high dose of 1000 mg/kg, EM induced an estrogen‐like effect when compared with the standard drug (estradiol valerat, a synthetic 17*β*‐estradiol). EM (1000 mg/kg) also significantly reduced (*P* < 0.05) serum levels of FSH and LH with no observable effects on estrogen and progesterone hormones (Table 5).

**Table 5:** Effect of EM on female reproductive hormonal levels

Hormones (mIU/mL)	Control	EM (100 mg/kg)	EM (1000 mg/kg)	Estrogen (100 mg/kg)	Progesterone (10 mg/kg)
FSH	2.93 ± 0.21	2.58 ± 0.22	1.83 ± 0.09*	1.73 ± 0.12*	2.78 ± 0.33
LH	3.75 ± 0.26	2.60 ± 0.25	1.93 ± 0.03**	2.33 ± 0.45*	2.78 ± 0.19
PROG	1.98 ± 0.13	0.85 ± 0.36*	1.17 ± 0.22	1.13 ± 0.09	1.20 ± 0.15
OEST	20.0 ± 0.57	20.4 ± 0.30	17.6 ± 0.33	18.0 ± 0.43	19.6 ± 0.73

Values are expressed as mean ± SEM (*n* = 5 mice). Control group received 0.5 mL/kg normal saline.

PROG, progesterone; OEST, estrogen. EM, *Emilia coccinea;* FSH, follicle‐stimulating hormone; LH, luteinizing hormone.

*P* ‐value *(*P* < 0.05) was considered to be statistically significant when compared with the control. ** *P* < 0.01.

### Effect of EM on the uterus and ovary

**Figure 2:**
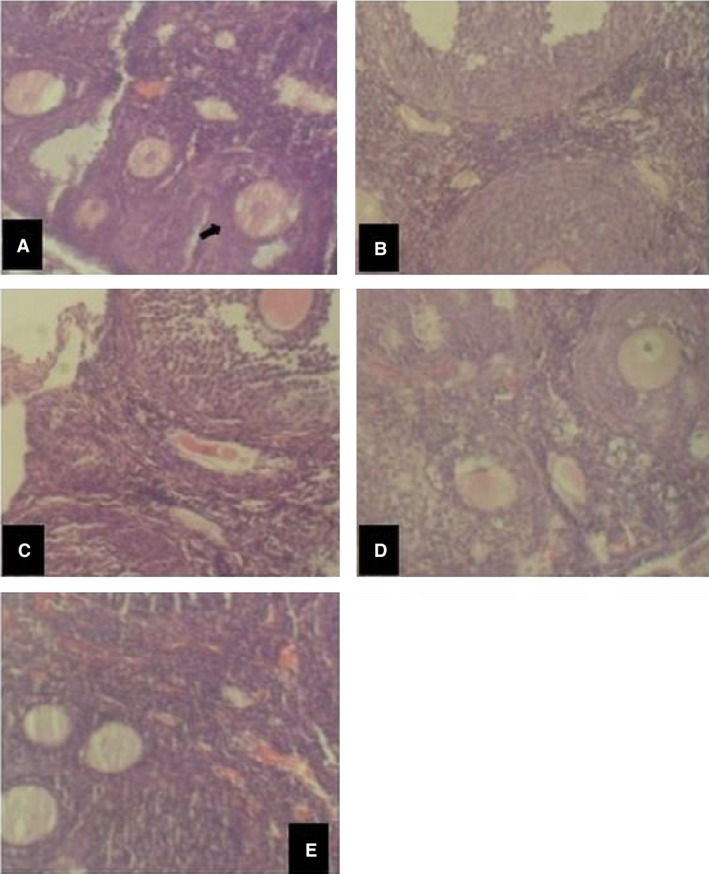
Representative images of hematoxylin and eosin staining of ovarian tissue from mice after 6 days of EM treatment. (A) Control (normal saline), (B) progesterone (10 mg/kg (C) Estrogen (100 mg/kg), (D) EM (1000 mg/kg), (E) EM (100 mg/kg). Follicles are indicated by arrow in (A). EM, *Emilia coccinea*.

**Figure 3:**
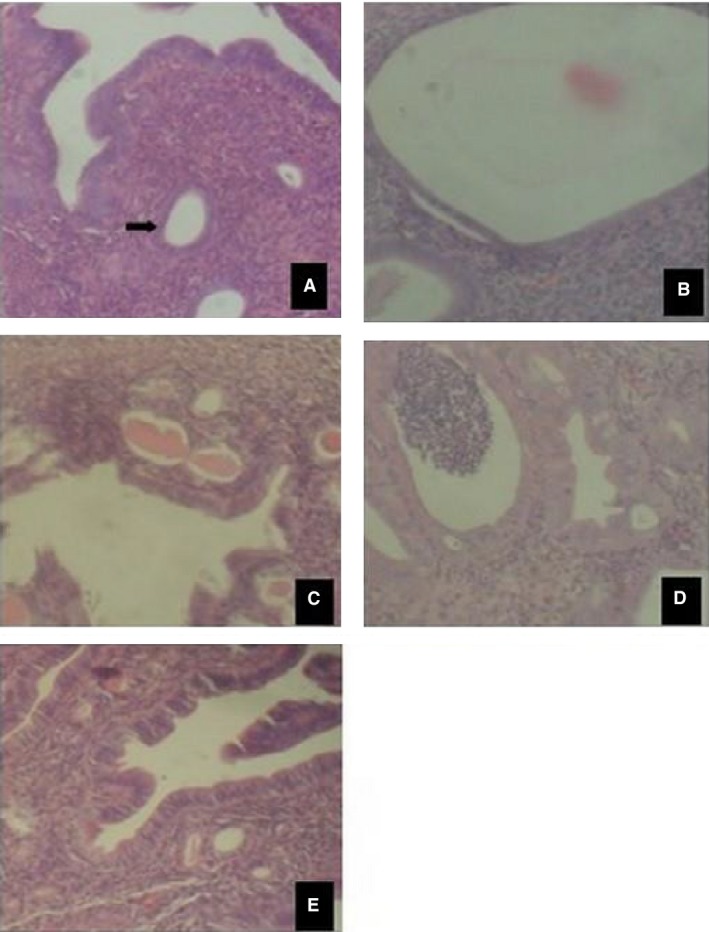
Mouse Uterus H & E ×100: Representative images of haematoxylin and eosin staining of uterine tissue from mice after 6 days of EM treatment. (A) Control (normal saline), (B) Estrogen (100 mg/kg), (C) Progesterone (10 mg/kg), (D) EM (1000 mg/kg), (E) EM (100 mg/kg). Uterine glands are indicated by arrow in (A). EM, *Emilia coccinea*.

The histological analysis of the uterus and ovary of EM‐treated mice showed that the high dose of EM (1000 mg/kg) exerted significant changes similar to the effects of the standard drug (estradiol valerat) (Figs. 2, 3) on both the tissues of the ovaries and uterus, whereas the low dose (100 mg/kg) showed mild changes in the structure of these tissues. The high dose EM caused maturation of ovarian follicles and luteinization of ovarian stroma as well as uterine cystic glandular hyperplasia with moderate luminal and stromal infiltrates of inflammatory cells.

### Identification of phytochemical constituents

**Figure 4:**
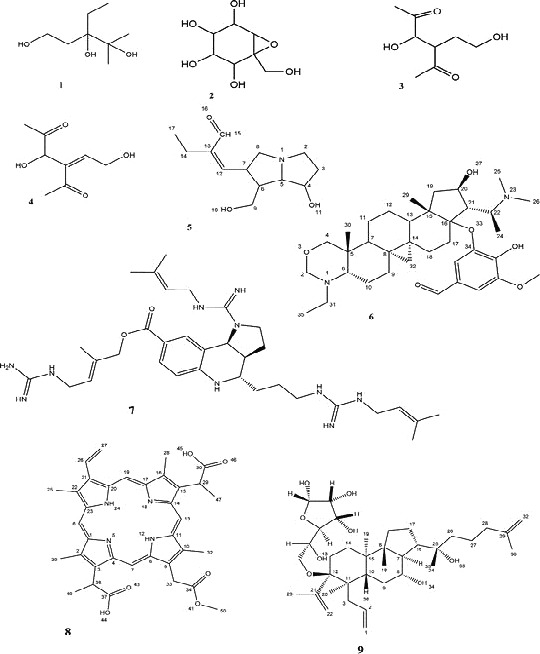
Structure for compound 9 needs to be changed.

The LC‐MS spectral results and database search enabled the detection of nine (9) major compounds (Table 6) and eleven (11) unidentified major compounds (Table 7) from the methanol crude extract (Tables 6 and 7). The unidentified compounds possibly represent new compounds and will require further purification procedures coupled with 1D and 2D NMR analyses for proper identification. The nine major compounds identified include: 3‐ethyl‐4‐methyl‐1,3,4‐pentanetriol (**1**), 5,6‐epoxy‐5‐(hydroxymethyl)‐1,2,3,4‐cyclohexanetetrol (**2**), 1,4‐butanediol Di‐Ac (**3**), 2‐butene‐1,4‐diol; (E)‐form (**4**), Di‐Ac, hexahydro‐7‐hydroxy‐1H‐pyrrolizine‐1‐methanol; (1S,7S,7aS)‐form, 1′‐angeloyl (**6**), cyclobuxoxazine C; 16‐O‐(4‐hydroxy‐3,5‐dimethoxybenzoyl) (**6**), martinelline (**7**), isoharderoporphyrin (**8**), 12‐O‐a‐L‐arabinofuranoside‐alnustic acid (**9**) (Table 6). The structures of identified compounds are shown in Figure 4.

**Table 6:** Putatively Identified Compounds in *Emilia coccinea*

	Compound Name	Molecular formula	Molecular weight (g/mol)	*m/z*	R_*t*_ (min)
1	3‐Ethyl‐4‐methyl‐1,3,4‐pentanetriol	C_8_H_18_O_3_	162.1256	[M+1]^+^ 163.1329	0.22
2	5,6‐Epoxy‐5‐(hydroxymethyl)‐1,2,3,4‐cyclohexanetetrol	C_7_H_12_O_6_	192.0635	[M−1]^−^191.0562	1.19
3	1,4‐Butanediol; Di‐Ac	C_8_H_14_O_4_	174.0891	[M−1]^−^ 173.0818	2.14
4	2‐Butene‐1,4‐diol; (E)‐form, Di‐Ac (possibly a fragment of compound **3**)	C_8_H_12_O_4_	172.0735	[M−1]^−^ 171.0663	2.28
5	Hexahydro‐7‐hydroxy‐1H‐pyrrolizine‐1‐methanol; (1S,7S,7aS)‐form, 1′‐Angeloyl	C_13_H_21_NO_3_	239.1519	[M+1]^+^ 240.1592	4.87
6	Cyclobuxoxazine C; 16‐O‐(4‐Hydroxy‐3,5‐dimethoxybenzoyl)	C_36_H_54_N_2_O_6_	610.3992	[M−1]^−^ 609.3919	25.05
7	Martinelline	C_33_H_52_N_10_O_2_	620.4283	[M−1]^−^ 619.4210	21.07
8	Isoharderoporphyrin	C_35_H_36_N_4_O_6_	608.2638	[M+1]^+^ 609.2710	24.93
9	Alnustic acid; 12‐O‐*α*‐L‐Arabinofuranoside	C_36_H_60_O_8_	620.4284	[M−1]^−^ 619.42115	35.33

**Table 7:** Unidentified compounds detected in *Emilia coccinea*. Double bond equivalence (DBE) indicates number of rings and double bonds in the structure where 1 ring = 1 DBE

	Double bond equivalence	Predicted formula	Molecular weight	*m/z*	*R*_*t*_
10	5.5	C_9_H_6_N_3_O_2_	188.0453	[M−1]^−^ 187.0381	1.14
11	8.0	C_29_H_40_N_4_O_7_	556.2911	[M−1]^−^ 555.2838	1.19
12	30.0	C_61_H_62_N_2_O	838.4888	[M−1]^−^ 837.4815	23.5
13	15.0	C_34_H_40_O_9_	592.2689	[M+1]^+^ 593.2763	29.33
14	6.0	C_29_H_48_O_2_	428.3652	[M+1]^+^ 429.3725	29.86
15	8.0 2.0	C_40_H_65_NO_3_ C_35_H_65_N_3_O_5_	607.4945	[M−1]^−^ 606.482	31.19
16	1.0 2.0	C_37_H_72_N_2_O_7_ C_38_H_68_N_6_O_3_	656.5343	[M−1]^−^ 655.5270	31.35
17	14.0	C_44_H_58_N_2_O_3_	662.4468	[M+1]^+^ 663.4541	35.74
18	13.0	C_44_H_61_N_3_O_3_	679.4736	[M+1]^+^ 680.4809	35.74
19	18.0	C_46_H_56_N_2_O_3_	684.4287	[M+1]^+^ 685.4360	35.78
20	7.0	C_36_H_55_N_5_O	573.4389	[M−1]^−^ 572.4315	35.87

**Figure 5:**
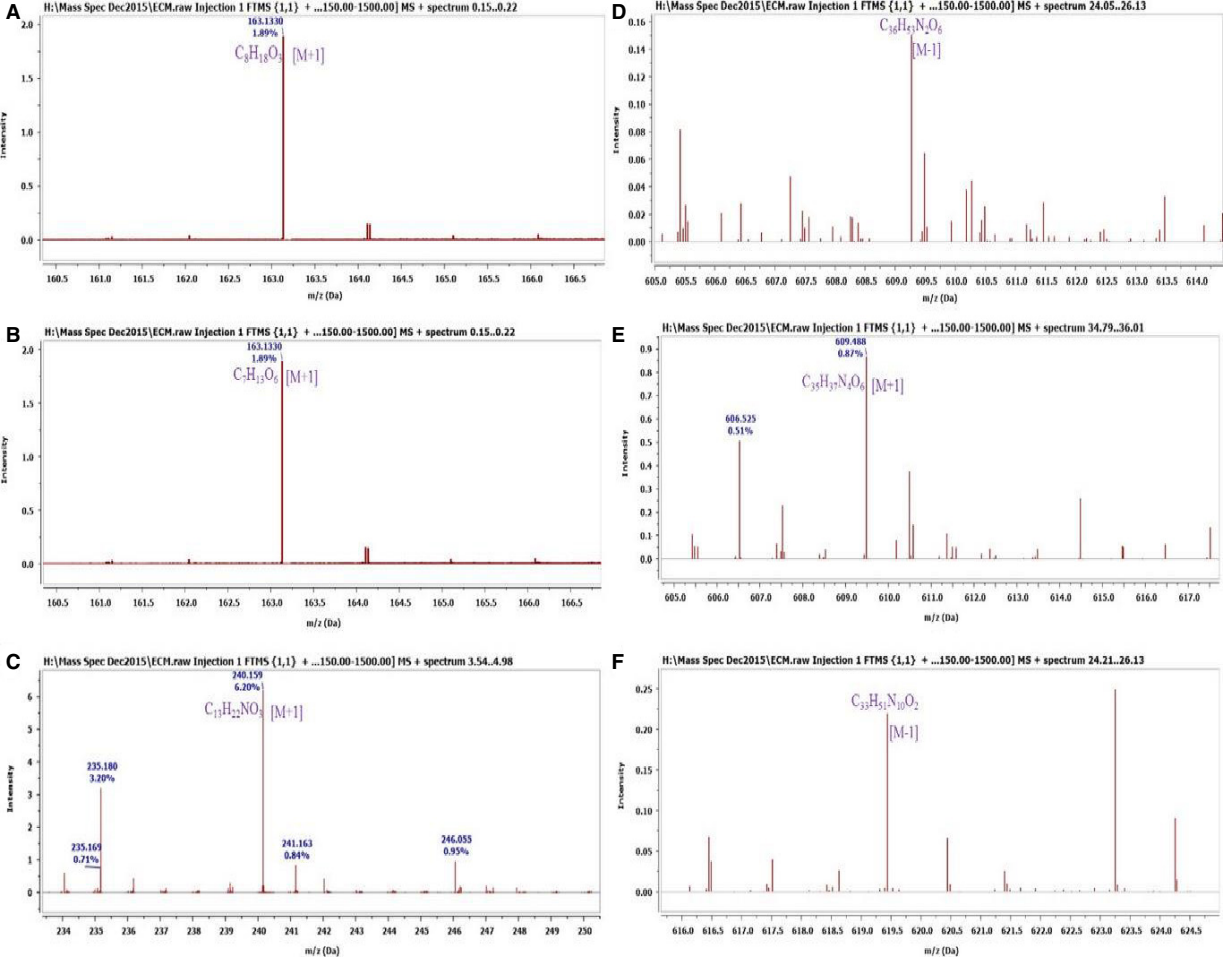
LC‐HRFTMS Chromatograms of selected identified compounds (Table 6) showing the mass to charge ratio (*m/z*) and the intensity. (A) represents the chromatogram of compound 1 with an intensity of 1.89%; (B) represents the chromatogram of compound 2 with an intensity of 1.89%; (C) represents the chromatogram of compound 5 with an intensity of 6.20%; (D) represents the chromatogram of compound 6 with an intensity of 0.16%; (E) represents the chromatogram of compound 7 with an intensity of 0.87%; (F) represents the chromatogram of compound 8 with an intensity of 0.23%.

Of the compounds identified, the LC‐HRFTMS chromatogram showed compound 5 to be the most intense compound present, whereas compound 6 was present in very low quantities (Fig. 5).

## Discussion

The result obtained from the acute toxicity studies showed a reasonable safety profile of the extract up to the dose of 2000 mg/kg after a 24 h test. Preclinical toxicity testing is one of the basic screening required to generate important information about potential damage of agents or new compounds to humans and is of utmost importance in drug development processes (Parasuraman 2011; David and Enegide, 2013). The US Food and Drug Administration states that the screening of new molecules for pharmacological activity and toxicity on animals is essential as this reveals similar incidence of occurrence and severity in humans (United States Code Sections, Statutes at Large, Public Laws, and Presidential Documents, Code 1992)^.^

The study of the effect of EM on the reproductive cycle revealed that high dose of EM prolonged the estrus cycle by prolonged or persistent estrus phase in mice. During normal estrus cycle of mice, increase in estrogen level coincides with proestrus phase and results in cornification of cells of the vaginal walls. Increased cornification up to 90% induces a new phase (estrus phase). At the onset of estrus, estrogen levels begin to drop with increase in levels of progesterone. This increase in progesterone levels leads to LH surge and consequently ovulation. Estrogen levels continue to fall and this leads to loss of cornification of vaginal cells and consequently, the next phase of the cycle (diestrus).

However, persistent estrus most often associated with antiovulatory effects of agents exhibited by high dose of EM indicated significant increase in estrogen levels which could not decline during the estrus phase. This shows that the antiovulatory effects of EM could be by mechanisms that either the block or prevent ovulation.

This report is consistent with previous studies of Huang and Meites (1975) and Brown‐Grant et al. (1973). They showed that an extended vaginal estrus often indicates that the female cannot spontaneously achieve the ovulatory surge of LH and that constant or persistent vaginal estrus (or cornification) may result if the vaginal epithelium becomes cornified and remains so in response to certain agents particularly those with estrogen‐like properties as found with diethylstilbesterol.

The clinical signs and symptoms of prolonged estrus include cornification of vaginal epithelial cells, tail flagging, and mating receptivity, as well as redness and swelling of the vulva (Eurell and Frappier 2006). Previous studies had shown that females in persistent or prolonged estrus may be sexually receptive regardless of the mechanisms underlying the altered ovarian condition (Smith and Davidson 1974; Cooper et al. 1993). However, Cooper et al. (1993) reported that if ovulation has been blocked by the treatment, LH surge may be induced by mating which would result in either pregnancy or pseudopregnancy but overall fertility of such mating is reduced (Fugo and Butcher 1966). They also showed that significant delays in ovulation can also result in increased embryonic abnormalities and pregnancy loss (Fugo and Butcher 1966; Kupfer 1987).

In this study, we compared the effects of different doses of EM, estradiol valerat, a synthetic 17*β*‐estradiol and progesterone on both uterine weight and histology of the uterus of mice to evaluate the estrogenic and antiestrogenic effects of EM. Results obtained showed that higher dose of EM induced significant uterotrophic effects similar to estradiol valerat, an estrogen receptor alpha (ER‐*α*) agonist.

To further evaluate the antifertility and possibly antiovulatory effects of EM, we evaluated the effects of EM on reproductive hormones and the histopathology of the ovaries. The extract at high dose was observed to reduce the levels of follicle‐stimulating hormone (FSH) and LH in the serum without significant alterations on the levels of estrogen and progesterone. And caused maturation of the follicles with no evidence of ovulation or formation of corpus lutea (postovulatory effects) indicating that ovulation never occurred.

Many authors have shown that monitoring estrogen and progesterone concentration in serum is not a reliable method for diagnosing cytological persistent estrus or antiovulatory effects as some species such as rodents and canines do not always show the normal increased levels of serum estrogen and progesterone (Groothuis et al. 2007; Nelson et al. 1981; Lu et al. 1979).

The increased uterine weight and decreased LH are fundamental responses of females to exposure of increased levels of estrogen and reduced levels of progesterone and can be as a result of the growth of glandular epithelium, increased blood vascularity and fluid accumulation or increase in expression of vascular endothelial growth factor (VEGF) (Kurniawan et al. 2014). Unlike the human uterus, endometrial growth in mice appears to be due to water imbibitions in the uterine lumina and in the tissue rather than cellular amplification (Goldman et al. 1990). This culminates into a hypertrophic response of the uterine tissues. In humans, estrogen dominance is a major cause of infertility in women (Navot and Bergh 1991).

The histological studies of the mouse uterus treated with both high dose of EM and high dose of estradiol valerate also revealed that both agents caused cystic glandular hyperplasia of uterine glands, a state of uncomplicated immoderate enlargement of endometrial glandular cells. This condition has been reported by many authors to result due to increased estrogen levels with accompanying very low levels of progesterone.

In rodents, the effects of exogenous estrogens on the uterus include hypertrophy of the luminal (predominantly) and glandular epithelium (Katsuda et al. 1999), proliferation of the myometrium (Hunter et al. 1999), and stroma (Cook et al. 1999). Several studies have confirmed that these effects are mediated through the estrogen receptor alpha (ER‐*α*) (Buchanan et al. 1999; Ogawa et al. 1999) suggesting that EM exerted its effects either by direct action on ER‐*α*, or it may be involved the mechanisms that might be unrelated to activation of ER‐*α* but by other mechanisms in which estrogen could be involved in.

This could possibly be by negative feedback decreases on the pulsatile frequency of GnRH release by the hypothalamus which decreases the release of FSH and LH by the anterior pituitary. The decreased levels of FSH inhibit further follicular development preventing any increase in estrogen levels. Then the insufficient levels of progesterone and lack of estrogen positive feedback on LH release prevent a mid‐cycle LH surge. This effect coupled with inhibition of further follicular development prevents ovulation.

This study correlates with several works such as that of Goldman et al. (1991) who reported that chemicals can delay or block ovulation by disrupting the ovulatory surge of LH, or by interfering with the ability of the maturing follicles to respond to the gonadotropic signals. Also, Pang et al. (1977) and Smith (1983) reported that compounds which increase central opioid receptor stimulation also decrease serum LH and inhibit ovulation in monkeys and rats. Further research on this aspect is, however, beyond the scope of this study and further studies is therefore advised.

### Chromatogram and biological effects of identified compounds

Four compounds observed in this study are likely implicated in the antifertility effects of EM. These include, (1S,7S,7aS)‐1′‐angeloyl hexahydro‐7‐hydroxy‐1H‐pyrrolizine‐1‐methanol; (compound 5), 3‐Ethyl‐4‐methyl‐1,3,4‐pentanetriol (compound 1), 5,6‐epoxy‐5‐(hydroxymethyl)‐1,2,3,4‐cyclohexanetetrol (compound 2), and isoharderoporphyrin (compound 8).

1′‐Angeloyl‐Hexahydro‐7‐hydroxy‐1H‐pyrrolizine‐1‐methanol; (1S,7S,7aS)‐form, identified by LC‐MS spectral belongs to the class of pyrrolizidine alkaloids (PAs). Some adverse effects which occur on consumption of plants have been associated with the PAs (Roeder 1995). PAs are secondary metabolites which have been known to occur mainly in the families *Asteraceae* (to which the EM in this study belongs), *Fabaceae*, and *Boraginaceae* (Roeder 1995). In particular, PAs have been identified in *Emilia* genus (Roeder 1995). PAs are generally known to exhibit toxic, carcinogenic, and mutagenic effects (Tidjani et al. 2013). Antifertility effects of PAs from *Senecio vulgaris* L. in rats have been reported (Tu et al. 1988). Similarly PAs from *Heliotropium Indicum* L. (Boraginaceae) have been reported to show significant antifertility, anti‐implantation and abortifacient activity in rats (Willaman and Schubert 1961; Das 2011). PAs have also been reported to show weak inhibition of intestinal and uterine smooth muscles (Akinlolu et al. 2006) but smooth muscle stimulatory effects have also been reported (Adelaja et al. 2008). The antifertility effects described for PAs and the identification of PAs in the *Emilia* genus strongly implicates compound 5 as a potential compound for further study as the active constituent responsible for the antifertility effects observed in this study.

Glycols have been reported to exhibit antitumor effects (Wang et al. 1996; Taketa et al. 2015), anxiolytic effects (Lin et al. 1998), and sedative effects in humans (Singh et al. 1981; Forrest and Galletly 1988). Reports have shown polyethylene glycol (PEG) to exert inhibitory effects on liver enzymes and on central nervous system activity (Nishimura et al. 1998; Thomsen et al. 1995). On effects on smooth muscle, PEG has been reported to exert an inhibitory effect on intestinal smooth muscles (Shah 1969), whereas propylene glycol has been reported to inhibit smooth muscle contractility of the uterus and intestines (Bonnardeaux 1971). Ethylene glycol has been reported to prevent reattachment of contraction filament crossbridges (Sakoda and Horiuti 1992) which would cause and sustain relaxation of smooth muscles. PEG‐cholesterol has also been reported to inhibit L‐type calcium channels (Ochi et al. 2014) an effect that may also contribute to the smooth muscle inhibiting effect. Glycol ethers are known to prolong gestation in animals possibly through inhibition of gap junctions (Marty and Loch‐Caruso 1998). These inhibitory effects on smooth muscles and particularly on uterine smooth muscles may contribute to the effect observed in this study. However, to the best of our knowledge there has been no previous reports on 3‐Ethyl‐4‐methyl‐1,3,4‐pentanetriol which is also a glycol, on uterine smooth muscle contractility.

Isoharderoporphyrin belongs to the porphyrin group of compounds to which heme and chlorophyll belong as well (Hendry and Jones 1980). A common precursor to heme and chlorophyll is protoporphyrin IX (Hendry and Jones 1980). Harderoporphyrin is an intermediate compound between coproporphyrin and protoporphyrin commonly found in animal tissues (Hendry and Jones 1980). However, a plant equivalent exists which is involved in chlorophyll synthesis and metabolism (Hendry and Jones 1980). Until recently, porphyrin derivatives from plants were not associated with biological effects besides effects on photodynamic tumor therapy (Nyman and Hynninen 2004; Pandey et al. 1991). Bafor and colleagues provided the first report on plant‐derived porphyrins on uterine contractility (Bafor et al. 2013) where it was reported that different porphyrin derivatives exhibited differing effects of stimulation and inhibition on uterine contractility (Bafor et al. 2013). This therefore suggests a role for plant‐derived porphyrin derivatives on reproductive function and possibly a role for compound 8 in the reproductive effects observed in this study.

Cyclohexanetetrol belongs to the oligosaccharide class of cyclitol carbohydrates or sugars (Ramanathan et al. 1966). Cyclitols which also include polyhydroxylated isocyclic molecules are involved in a number of biological functions often coupled to other functional groups (Lehn et al. 2007). Inositols which are also cyclitols and are referred to as polyhydroxylated cyclohexane derivatives with myo‐inositol being the most naturally abundant, also play significant biological roles (Lehn et al. 2007). There has, however, been no report on cyclohexanetetrol or similar nonphosphorylated cyclitol derivative on smooth muscle contractility or signaling. Further studies are therefore required. It is, however, suggested that cyclohexanetetrol may function as a precursor to phosphorylated inositols.

In conclusion, this study has established potential antifertility activities of the methanol leaf extract of *Emilia coccinea* Sims (G) Dons. However, further research is advised to fully establish the mechanism of the active constituent of this plant.

## Conflict of Interests

The authors declare no conflict of interest.

## References

[phy213047-bib-0001] Addae‐Mensah, I . 1999 Towards a Rational Science Basis for Herbal Medicine Pp 63–65 *in* A phytochemist two decades contribution. Ghana University Press, Accra.

[phy213047-bib-0002] Adelaja, A. A. , M. D. Ayoola , J. O. Otulana , O. B. Akinola , A. Olayiwola , and A. B. Ejiwunmi . 2008 Evaluation of the histo‐gastroprotective and antimicrobial activities of Heliotropium indicum Linn (Boraginaceae). Malay. J. Med. Sci. 15:22. PMC3341904 22570586

[phy213047-bib-0003] Agoha, R. C . 1981 Medicinal plants of Nigeria. *Offsetdikker Jifaculteit waskunden, Natnurwenten schopp; pen, Netherlands*, 22.158.

[phy213047-bib-0004] Akinlolu, A. A. , M. Sadiq , M. D. Ayoola , J. O. Otulana , O. Abimbola , and A. B. Ejiwunmi . 2006 Morphological gastroprotective effects of Heliotropium indicum on gastric ulcerated mucosa. Pak. J. Path. 17:60–64.

[phy213047-bib-0005] Bafor, E. E. , C. V. Lim , E. G. Rowan , and R. Edrada‐Ebel . 2013 The leaves of Ficus exasperata Vahl (Moraceae) generates uterine active chemical constituents. J. Ethnopharmacol. 145:803–812. 23266275 10.1016/j.jep.2012.12.020

[phy213047-bib-0006] Bonnardeaux, J. L. 1971 A comparison of the effects of three organic solvents: dimethyl sulfoxide, formamide, and propylene glycol, on spontaneous activity of isolated smooth muscle. Can. J. Physiol. Pharmacol. 49:632–641. 5141681 10.1139/y71-085

[phy213047-bib-0007] Brown‐Grant, K. , J. M. Davidson , and F. Grieg . 1973 Induced ovulation in albino rats exposed to constant light. J. Endocrinol. 57:7–22. 4735633 10.1677/joe.0.0570007

[phy213047-bib-0008] Buchanan, D. L. , T. Sctiawan , D. B. Lubahn , J. A. Taylor , T. Kurita , G. R. Cunha , et al. 1999 Tissue compartment‐estrogen receptor‐*α* participation in the mouse uterine epithelial secretory response. Endocrinology 140:484–491. 9886861 10.1210/endo.140.1.6448

[phy213047-bib-0009] Burkill, H. M. 1984 The useful plants of West Tropical Africa. Families J‐L. Royal Botanical Garden kew 3:522.

[phy213047-bib-0010] Chillendon, F . 1956 Pp. 113–115 *in* Dictionary of plant plus supplements. Oxford University Press, London.

[phy213047-bib-0011] Cook, J. C. , M. Kaplan , L. G. Davis , and J. C. O'Connor . 1999 Development of a Tier I screening battery for detecting endocrine‐active compounds (EACs). Reg. Toxicol. Pharmacol. 26:60–68. 10.1006/rtph.1997.1120 9339481

[phy213047-bib-0012] Cooper, R. L. , J. M. Goldman , and J. G. Vandenbergh . 1993 Monitoring of the estrous cycle in the laboratory rodent by vaginal lavage Pp. 45–56 *in*: Heindel J. J., Chapin R. E., eds. Methods in toxicology: female reproductive toxicology. Academic Press, San Diego.

[phy213047-bib-0013] Cunningham, A. B . 1993 African medicinal plants: setting priorities at the Interface between conservation and primary healthcare Pp 92 *in* People and Plants Working paper I. UNESCO, Paris.

[phy213047-bib-0014] Das, P. K. 2011 Antibacterial activity of leaf extracts of Heliotropium indicum Linn. Life Sci. Leaflets 20:904–907.

[phy213047-bib-0015] David and Enegide . 2013 The importance of toxicity testing. J. Pharm. Bio. Sci. 4:146–148.

[phy213047-bib-0016] Edeoga, H. O. , D. E. Okwu , and B. O. Mbaebie . 2005 Phytochemical constituents of some Nigerian medicinal plants. Afr. J. Biotechnol. 4:685–688.

[phy213047-bib-0017] Elvis‐Offiah, U. B. , and E. E. Bafor . 2014 Evaluation of the effects of oxytocin and diethylstilboesterol on mouse oestrous cycle using an index. JMBR 13:5–16.

[phy213047-bib-0018] Erhabor, J. O. , E. O. Oshomoh , O. Timothy , E. S. Osazuwa , and M. Idu . 2013 Antimicrobial activity of the methanol and aqueous leaf extracts of *Emilia coccinea* (Sims) G Don. Nig. J. Biotech. 25:37–45.

[phy213047-bib-0019] Eurell, J. A. , and B. L. Frappier . 2006 Female Reproductive System Pp. 256–278 In Dellmann's Textbook of Veterinary Histology. Iowa, USA, Blackwell publishing, Sixthed. Ames.

[phy213047-bib-0020] Forrest, P. , and D. C. Galletly . 1988 A double‐blind comparative study of three formulations of diazepam in volunteers. Anaesth. Intensive Care 16:158–163. 3394908 10.1177/0310057X8801600205

[phy213047-bib-0021] Foyet, H. S. , B. A. Abdou , A. H. Ngatanko , F. L. Manyi , N. A. Manyo , P. N. Shu , et al. 2014 Neuroprotective and memory improvement effects of a standardized extract of Emilia coccinea (SIMS) G. on animal models of anxiety and depression. J. Pharmacogn. Phytochem. 3: 146–154.

[phy213047-bib-0022] Fugo, N. W. , and R. L. Butcher . 1966 Overripeness and the mammalian ova. I. Overripeness and early embryonic development. Fertil. Steril. 17:804–814. 5950753 10.1016/s0015-0282(16)36132-5

[phy213047-bib-0023] Goldman, J. M. , R. L. Cooper , S. C. Laws , G. L. Rehnberg , T. L. Edwards , W. K. McElroy , et al. 1990 Chlordimeform‐induced alterations in endocrine regulation within the male rat reproductive system. Toxicol. Appl. Pharmacol. 104:25–35. 2113720 10.1016/0041-008x(90)90279-4

[phy213047-bib-0024] Goldman, J. M. , R. L. Cooper , T. L. Edwards , G. L. Rehnberg , W. K. McElroy , and J. F. Hein . 1991 Suppression of the luteinizing hormone surge by chlordimeform in ovariectomized, steroid‐primed female rats. Pharmacol. Toxicol. 68:131–136. 1852718 10.1111/j.1600-0773.1991.tb02050.x

[phy213047-bib-0025] Groothuis, P. G. , H. H. N. M. Dassen , A. Romano , and C. Punyadeera . 2007 Estrogen and the endometrium: lessons learned from gene expression profiling in rodents and human. Human Reprod. Update 13:405–417. 10.1093/humupd/dmm009 17584823

[phy213047-bib-0026] Hendry, G. A. , and O. T. Jones . 1980 Haems and chlorophylls: comparison of function and formation. J. Med. Genet. 17:1–14. 6988593 10.1136/jmg.17.1.1 PMC1048480

[phy213047-bib-0027] Hewitt, S. C. , B. J. Deroo , K. Hansen , J. Collins , S. Grissom , C. A. Afshari , et al. 2003 Estrogen receptor‐dependent genomic responses in the uterus mirror the biphasic physiological response to estrogen. Mol. Endocrinol. 17:2070–2083. 12893882 10.1210/me.2003-0146

[phy213047-bib-0028] Hewitt, S. C. , J. Collins , S. Grissom , B. Deroo , and K. S. Korach . 2005 Global uterine genomics in vivo: microarray evaluation of the estrogen receptor alpha‐growth factor cross‐talk mechanism. Mol. Endocrinol. 19:657–668. 15528273 10.1210/me.2004-0142

[phy213047-bib-0029] Huang, H. H. , and J. Meites . 1975 Reproductive capacity of aging female rats. Neuroendocrinology 17:289–295. 1170518 10.1159/000122367

[phy213047-bib-0030] Hunter, D. S. , L. C. Hodges , P. M. Vonier , R. Fush‐young , M. M. Gottardis , and C. I. Walker . 1999 Estrogen receptor activation via activation function 2 predict agonism of xenoestrogens in normal and neoplastic cells of the uterine myometrium. Cancer Res. 59:3090–3099. 10397250

[phy213047-bib-0031] Idu, M. , J. O. Erhabor , O. Timothy , and S. O. Etatuvie . 2010 Phytochemical and acute toxicity studies of the aqueous and methanol extracts of *Emilia coccinea* (Sims) G Don. J. Plant Developmental Sci. 2(3&4):89–94.

[phy213047-bib-0032] Jendrassik, L. , and P. Grof . 1938 Colorimetric method of determination of bilirubin. Biochem. J. 297:81–82.

[phy213047-bib-0033] Kaplan, M. 1972 Alkaline phosphatase. Gastroenterology 62:452–465. 4551808

[phy213047-bib-0034] Katsuda, S. I. , M. Yoshida , T. Watanabe , H. Kuroda , J. Ando‐Lu , M. Takahashi , et al. 1999 Estrogen receptor mRNA in uteri of normal estrous cycling and ovariectomize rats by insitu hybridization. Proc. Soc. Exp. Biol. Med. 221:207–214. 10404038 10.1046/j.1525-1373.1999.d01-78.x

[phy213047-bib-0035] Kessner, D. , M. Chambers , R. Burke , D. Agus , and P. Mallick . 2008 Open source software for rapid proteomics tools development bioinformatics doi: 10.1093/bioinformatics/btn323 10.1093/bioinformatics/btn323 PMC2732273 18606607

[phy213047-bib-0036] Kubmarawa, D. , G. A. Ajoku , N. M. Enwerem , and D. A. Okorie . 2007 Preliminary phytochemical and antimicrobial screening of 50 medicinal plants from Nigeria. Afr. J. Biotechnol. 6:1690–1696.

[phy213047-bib-0037] Kupfer, D. 1987 Critical evaluation of methods for detection and assessment of estrogenic compounds in mammals: strengths and limitations for application to risk assessment. Reprod. Toxicol. 2:147–153. 10.1016/0890-6238(87)90010-4 2980373

[phy213047-bib-0038] Kurita, T. , R. Medina , A. B. Schabel , P. Young , P. Gama , T. V. Parekh , et al. 2005 The activation function‐1 domain of estrogen receptor alpha in uterine stromal cells is required for mouse but not human uterine epithelial response to estrogen. Differentiation 73:313–322. 16138832 10.1111/j.1432-0436.2005.00033.x

[phy213047-bib-0039] Kurniawan, E. , T. Djuwantono , U. Sabarudin , S. R. Krisnadi , W. Permadi , and T. H. Madjid . 2014 Difference of endometrial thickness and vascularity in women stimulated by clomiphene citrate with and without vitamin C and E. Am. J. Res.Commun. 2:1–10.

[phy213047-bib-0040] Lehn, J. M. , S. Pothukanuri , A. Koumbis , C. Duarte , and C. Nicolau . 2007.U.S. Patent Application No. 11/967,424.

[phy213047-bib-0041] Lin, H. Q. , P. M. Burden , and G. A. Johnston . 1998 Neuropharmacology: Propylene Glycol Elicits Anxiolytic‐like Responses to the Elevated Plus‐maze in Male Mice. J. Pharm. Pharmacol. 50:1127–1131. 9821659 10.1111/j.2042-7158.1998.tb03323.x

[phy213047-bib-0042] Lu, K. H. , B. R. Hopper , T. M. Vargo , and S. S. Yen . 1979 Chronological changes in sex steroid, gonadotropin, and prolactin secretion in ageing female rats displaying different reproductive states. Biol. Reprod. 21:193–203. 573635 10.1095/biolreprod21.1.193

[phy213047-bib-0043] Marty, M. S. , and R. Loch‐Caruso . 1998 2‐Methoxyethanol inhibits gap junctional communication in rat myometrial myocytes. Cell Biol. Toxicol. 14:199–210. 9689493 10.1023/a:1007414610681

[phy213047-bib-0044] Mensah, J. K. , J. Ihenyen , and M. Iyamu . 2013 Phytochemical and antimicrobial properties of *Emilia coccinea* (cass.). Asian J. Contemp. Sci. 2:26–31.

[phy213047-bib-0045] Miller, L. C. , and M. L. Tainter . 1944 Estimation of the ED_50_ and its errors by means of logarithmic‐probit graph paper. Proc. Soc. Exp. Biol. Med. 57:261–264.

[phy213047-bib-0046] Navot, D. , and P. Bergh . 1991 Preparation of the human endometrium for implantation. Ann. NY Acad. Sci. 622:212–219. 2064182 10.1111/j.1749-6632.1991.tb37864.x

[phy213047-bib-0047] Nelson, J. F. , L. S. Felicio , H. H. Osterburg , and C. E. Finch . 1981 Altered profiles of estradiol and progesterone associated with prolonged estrous cycles and persistent vaginal cornification in aging C57bl/6J mice. Biol. Reprod. 24:784–794. 7195743 10.1095/biolreprod24.4.784

[phy213047-bib-0048] Nishimura, Y. , N. Kurata , M. Iwase , H. Li , and H. Yasuhara . 1998 The effects of organic solvents on trimethadione n‐demethylation in rats. Res. Commun. Mol. Pathol. Pharmacol. 104:229–239. 10634315

[phy213047-bib-0049] Nyman, E. S. , and P. H. Hynninen . 2004 Research advances in the use of tetrapyrrolic photosensitizers for photodynamic therapy. J. Photochem. Photobiol., B 73:1–28. 14732247 10.1016/j.jphotobiol.2003.10.002

[phy213047-bib-0050] Ochi, R. , S. Chettimada , and S. A. Gupte . 2014 Poly (ethylene glycol)‐cholesterol inhibits L‐Type Ca 2 + channel currents and augments voltage‐dependent inactivation in A7r5 Cells. PLoS ONE 9:e107049. 25197984 10.1371/journal.pone.0107049 PMC4157810

[phy213047-bib-0051] Odugbemi, T. 2006 Pp. 283–285 *In* Outlines and pictures of Medicinal plants from Nigeria. University of Lagos press, Nigeria.

[phy213047-bib-0052] Ogawa, S. , J. Chan , A. E. Chester , J. A. Gustafsson , K. S. Korach , and D. W. Pfaff . 1999 Survival of reproductive behaviours in estrogen receptor beta gene‐deficient (beat ERKO) male and female mice. Proc. Natl Acad. Sci. 96:12887–12892. 10536018 10.1073/pnas.96.22.12887 PMC23148

[phy213047-bib-0053] Ogunlesi, M. , W. Okiei , and M. Ademoye . 2008 Medicinal plants used in treating eye infections in Nigeria Pp 299–317. In A Textbook of Medicinal plants from Nigeria. University of Lagos press, Nigeria.

[phy213047-bib-0054] Okiei, W. , M. Ogunlesi , and M. A. Ademoye . 2009 An assessment of the antimicrobial properties of extracts of various polarities from *Chasmanthera dependens*,* Emilia coccinea* and *Cuscuta australis*, herbal medications for eye diseases. J. Appl. Sci. 9:4076–4080.

[phy213047-bib-0055] Okoegwale, E. E. , and G. O. Olumese . 2001 Folk medicine practice in Nigeria, some medicinal plant of Esan people in Edo state, Nigeria. Nigeria Journal of Applied Science 4:2350–2360.

[phy213047-bib-0056] Okoegwale, E. E. , and J. U. Omefezi . 2001 Some herb preparation among the people of Isoko clan of Delta State, Nigeria. Niger. J. Applied Sci. 4:2359–2371.

[phy213047-bib-0057] Olorode, O. 1984 Pp445 *in* Taxomony of West Africa flowering plants. Longman, London

[phy213047-bib-0058] Pandey, R. K. , D. A. Bellnier , K. M. Smith , and T. J. Dougherty . 1991 Chlorin and porphyrin derivatives as potential photosensitizers in photodynamic therapy. Photochem. Photobiol. 53:65–72. 2027908 10.1111/j.1751-1097.1991.tb08468.x

[phy213047-bib-0059] Pang, C. N. , E. Zimmerman , and C. H. Sawyer . 1977 Morphine inhibition of preovulatory surges of plasma luteinizing hormone and follicle stimulating hormone in the rat. Endocrinology 101:1726–1732. 338290 10.1210/endo-101-6-1726

[phy213047-bib-0060] Parasuraman, S. 2011 Toxicological screening. J Pharmacol. Pharmacother. 2:74–79. 21772764 10.4103/0976-500X.81895 PMC3127354

[phy213047-bib-0061] Pluskal, T. , S. Castillo , A. Villar‐Briones , and M. Orešič . 2010 MZmine 2: modular framework for processing, visualizing, and analyzing mass spectrometry‐based molecular profile data. BMC Bioinformatics 11:1. 20650010 10.1186/1471-2105-11-395 PMC2918584

[phy213047-bib-0062] Ramanathan, J. D. , J. S. Craigie , J. McLachlan , D. G. Smith , and A. G. McInnes . 1966 The occurrence of D‐(+)‐1425‐cyclohexanetetrol in monochrysislutheri droop. Tetrahedron Lett. 7:1527–1531.

[phy213047-bib-0063] Reitman, S. , and S. Frankel . 1957 Glutamic – pyruvate transaminase assay by colorimetric method. Am. J. Clin. Path. 28:56. 13458125 10.1093/ajcp/28.1.56

[phy213047-bib-0064] Roeder, E. 1995 Medicinal plants in Europe containing pyrrolizidine alkaloids. Pharmazie 50:83–98. 7700976

[phy213047-bib-0065] Sakoda, T. , and K. Horiuti . 1992 Effects of ethylene glycol and calcium on the kinetics of contraction induced by photo‐release of low concentrations of ATP in rat psoas muscle fibres. J. Muscle Res. Cell Motil. 13:464–472. 1401042 10.1007/BF01738041

[phy213047-bib-0066] Shah, D. S. 1969 Effects of propylene glycol on the intestinal smooth muscles of experimental animals. Indian J. Physiol. Pharmacol. 13:147–151. 5362629

[phy213047-bib-0067] Singh, P. P. , A. Y. Junnarkar , C. Seshagirirao , R. Kaushal , M. U. Naidu , R. K. Varma , et al. 1981 A pharmacological study of propane‐1, 2‐diol. Arzneimittelforschung 32:1443–1446. 6891251

[phy213047-bib-0068] Smith, C. G. 1983 Reproductive toxicity: hypothalamic‐pituitary mechanisms. Am. J. Ind. Med. 4:107–112. 6301271

[phy213047-bib-0069] Smith, E. R. , and J. M. Davidson . 1974 Luteinizing hormone releasing factor in rats exposed to constant light: effects of mating. Neuroendocrinology 14:129–138. 4604709 10.1159/000122253

[phy213047-bib-0070] Snell G. D., ed. 1941 Biology of the laboratory mouse. The Blakiston Company, Philadelphia.

[phy213047-bib-0071] Sofowora, E. A. 1982 Pp198 *in* Medicinal plants and traditional medicine in Africa. John Wiley and sons, Chiclester.

[phy213047-bib-0072] Sofowora, E. A. 1993 Pp289 *in* Medicinal plants and traditional medicine in Africa. Spectrum Boot Ltd., Ibadan, Nig.

[phy213047-bib-0073] Stockard, C. R. , and C. N. Papanicolaou . 1917 The existence of a typical oestrous cycle in the guinea‐pig with a study of its histological and physiological changes. Am. J. Anat. 22:225–283.

[phy213047-bib-0074] Taketa, Y. , K. Inoue , M. Takahashi , Y. Sakamoto , G. Watanabe , K. Taya , et al. 2015 Effects of sulpiride and ethylene glycol monomethyl ether on endometrial carcinogenicity in Donryu rats. J. Appl. Toxicol. 36:769–776. 26178146 10.1002/jat.3206

[phy213047-bib-0075] Teke, G. N. , J. R. Kuiate , O. B. Ngouateu , and D. Gatsing . 2007 Antidiarrhoeal and antimicrobial activities of *Emilia coccinea* (Sims) G. Don extracts. J. Ethnopharmacol. 112:278–283. 17433589 10.1016/j.jep.2007.03.007

[phy213047-bib-0076] Telefo, P. B. , L. L. Lienou , M. D. Yemele , M. C. Lemfack , C. Mouokeu , C. S. Goka , et al. 2011 Ethnopharmacological survey of plants used for the treatment of female infertility in Baham, Cameroon. J. Ethnopharmacol. 136:178–187. 21540100 10.1016/j.jep.2011.04.036

[phy213047-bib-0077] Thomsen, M. S. , S. Loft , D. W. Roberts , and H. E. Poulsen . 1995 Cytochrome P4502E1 inhibition by propylene glycol prevents acetaminophen (paracetamol) hepatotoxicity in mice without cytochrome P4501A2 inhibition. Pharmacol. Toxicol. 76:395–399. 7479582 10.1111/j.1600-0773.1995.tb00168.x

[phy213047-bib-0078] Tidjani, S. , P. N. Okusa , A. Zellagui , L. M. Banuls , C. Stevigny , P. Duez , et al. 2013 Analysis of pyrrolizidine alkaloids and evaluation of some biological activities of Algerian Senecio delphinifolius (Asteraceae). Nat. Product Commun. 8:439–440. 23738446

[phy213047-bib-0079] Tu, Z. B. , C. Konno , D. D. Soejarto , D. P. Waller , A. S. Bingel , R. J. Molyneux , et al. 1988 Identification of senecionine and senecionine N‐oxide as antifertility constituents in Senecio vulgaris. J. Pharm. Sci. 77:461–463. 3411472 10.1002/jps.2600770522

[phy213047-bib-0080] United States Code Sections, Statutes at Large, Public Laws, and Presidential Documents, Code . 1992 Food and Drugs: 21 CER part 314‐Application for FDA Approval to market a new drug.

[phy213047-bib-0081] Wang, L. , Y. Wu , and Y. Zhang . 1996 In vivo antitumor effects of polyethylene glycol–modified recombinant human interleukin‐2 on mouse uterine cervical carcinoma. Zhonghua zhong liu za zhi [Chinese journal of oncology] 18:253–255. 9387313

[phy213047-bib-0082] Willaman, J. J. , and B. G. Schubert . 1961 Alkaloid‐bearing plants and their contained alkaloids (No. 1234). Agricultural Research Service, US Department of Agriculture.

[phy213047-bib-0083] Yolken, R. H. , and P. J. Stopa . 1979 Enzyme‐linked FLUORESCENCE assay: ultrasensitive solid‐phase assay for detection of human rotavirus. J. Clin. Microbiol. 10:317–321. 226564 10.1128/jcm.10.3.317-321.1979 PMC273160

[phy213047-bib-0084] Young, D. S. , L. C. Pestaner , and V. Gibberman . 1975 Effects of drugs on clinical laboratory tests. Clin. Chem. 21:1D–432D. 1091375

