# Erratum

**DOI:** 10.14814/phy2.13147

**Published:** 2017-02-13

doi: 10.14814/phy2.13147

In Elvis‐Offiah et al. (2016), the following error was published in the article.

The legend of Figure 4 reads:

“Structure for compound 9 needs to be changed.”

However, it should instead read:

“Compounds identified from *Emilia coccinea*.”

We apologize for the error.
